# The Self-Aggregation of Porphyrins with Multiple Chiral Centers in Organic/Aqueous Media: The Case of Sugar- and Steroid-Porphyrin Conjugates

**DOI:** 10.3390/molecules25194544

**Published:** 2020-10-04

**Authors:** Manuela Stefanelli, Federica Mandoj, Gabriele Magna, Raffaella Lettieri, Mariano Venanzi, Roberto Paolesse, Donato Monti

**Affiliations:** 1 Dipartimento di Scienze e Tecnologie Chimiche, Università di Roma Tor Vergata, via della Ricerca Scientifica, 1, 00133 Rome, Italy; federica.mandoj@uniroma2.it (F.M.); gabriele.magna@uniroma2.it (G.M.); raffaella.lettieri@uniroma2.it (R.L.); venanzi@uniroma2.it (M.V.); roberto.paolesse@uniroma2.it (R.P.); dmonti@uniroma2.it (D.M.); 2 Dipartimento di Chimica, Università di Roma Sapienza, P.le A. Moro 5, 00185 Rome, Italy

**Keywords:** porphyrins, self-aggregation, solvodichroic effect, chirality, circular dichroism, nanostructures, supramolecular chemistry, steroid derivatives, glycosylated compounds

## Abstract

An overview of the solvent-driven aggregation of a series of chiral porphyrin derivatives studied by optical methods (UV/Vis, fluorescence, CD and RLS spectroscopies) is herein reported. The investigated porphyrins are characterized by the presence in the meso-positions of glycol-, steroidal- and glucosteroidal moieties, conferring amphiphilicity and solubility in aqueous media to the primarily hydrophobic porphyrin platform. Aggregation of the macrocycles is driven by a change in bulk solvent composition, forming architectures with supramolecular chirality, steered by the stereogenic centers on the porphyrin peripheral positions. The aggregation behavior and chiroptical properties of the final aggregated species strongly depend on the number and stereogenicity of the ancillary groups that dictate the mutual spatial arrangement of the porphyrin chromophores and their further organization in larger structures, usually detectable by different microscopies, such as AFM and SEM. Kinetic studies are fundamental to understand the aggregation mechanism, which is frequently found to be dependent on the substrate concentration. Additionally, Molecular Mechanics calculations can give insights into the intimate nature of the driving forces governing the self-assembly process. The critical use of these combined methods can shed light on the overall self-assembly process of chirally-functionalized macrocycles, with important implications on the development of chiral porphyrin-based materials.

## 1. Introduction

Porphyrins are among the most versatile macrocycles fulfilling vital functions in living systems as well as practical applications including materials science, sensing, photovoltaics or medical treatments [1,2,3,4]. Although it is true that monomeric porphyrins possess valuable properties, such as catalytic [5] or sensitizers for dye-sensitized solar cells (DSCCs) [6], their self-assembly in sophisticated, size and shape-controlled suprastructures often significantly boosts their potentialities, leading to advanced functional materials with improved properties [7,8,9,10]. Accordingly, the achievement of these soft materials with desired functions represents an ongoing challenge for many researchers that continuously report on novel preparation protocols and characterization techniques up to their final applicative goal [11,12].

Furthermore, the implementation of elements of chirality on such systems widens their applicability in different fields of science and technology, spanning from asymmetric catalysis to chiral sensing [13,14]. Chiral porphyrin-based architectures can be obtained from either chiral or achiral platforms [11,15,16,17]. If in the former case the stereochemical course of the self-assembly process is dictated by the chiral functionalities anchored on the periphery of the macrocycle [18,19], in the other, the interaction of achiral monomers with chiral external effectors, such as hydrodynamic directional forces [20,21], magnetic fields [22], or chiral molecular templates [23,24,25,26,27,28,29] drives the formation of specific suprastructures with chiroptical properties. The literature has an abundance of examples that use both approaches to constructing chiral porphyrin assemblies, which, taken together, evidence the complexity and the variety of experimental factors determining the supramolecular chirality of the final systems both in solution [11] and in the solid state [30].

For many years, we have focused on the possibility to build up chiral porphyrin suprastructures by assembling inherently chiral porphyrin derivatives having single or multiple stereogenic centers peripherally located on the macrocycle thanks to the conjugation with diverse biomolecules, as amino acids, glucosides or steroids [31,32,33,34,35,36,37,38,39,40,41,42]. The self-aggregation of these compounds was induced in organic/aqueous mixtures by the so-called “good-bad” solvent protocol, in which porphyrins solubilization in a “good” solvent is followed by the addition of the proper amount of a “bad” one, where typically the macrocycle is insoluble, even at low concentrations. In all investigated systems, the self-assembly process is driven by the stereogenic groups that dictate the supramolecular chiral features of the formed non-covalent architectures.

In this contribution, we report the comprehensive results we gained in the last decade by investigating the solvent-promoted aggregation of porphyrin functionalized with a robust *C*-glycosidic group, a steroid or glucosylated steroid moieties, respectively. The formation of the aggregated species in aqueous solvent mixtures was studied by spectroscopic, morphological and mechanistic point of view, revealing interesting connections between the type of stereogenic information borne by the porphyrin macrocycle and the resulting aggregates with specific alignment and supramolecular chirality.

## 2. The Solvent-Promoted Aggregation of Chiral Porphyrin Derivatives: Our General Approach

Porphyrins are hydrophobic macrocycles, generally soluble in organic solvents of different polarity depending on the functional groups that decorate their periphery. The introduction of charged or polar functionalities into the porphyrin frame confers an amphiphilic character to the macrocycle that can be exploited to form aggregated structures in organic-water media by finely controlling experimental parameters. The identification of the best bulk solvent conditions (i.e., type and ratio between organic solvent and water, concentration and ionic strength) is strictly related to the porphyrin structural features, in particular to the subtle balance between the polar, chiral-appended functionality and the hydrophobic tetrapyrrolic platform, which together determine the extent of the overall solvophilicity of the porphyrin derivative.

**Figure 1:**
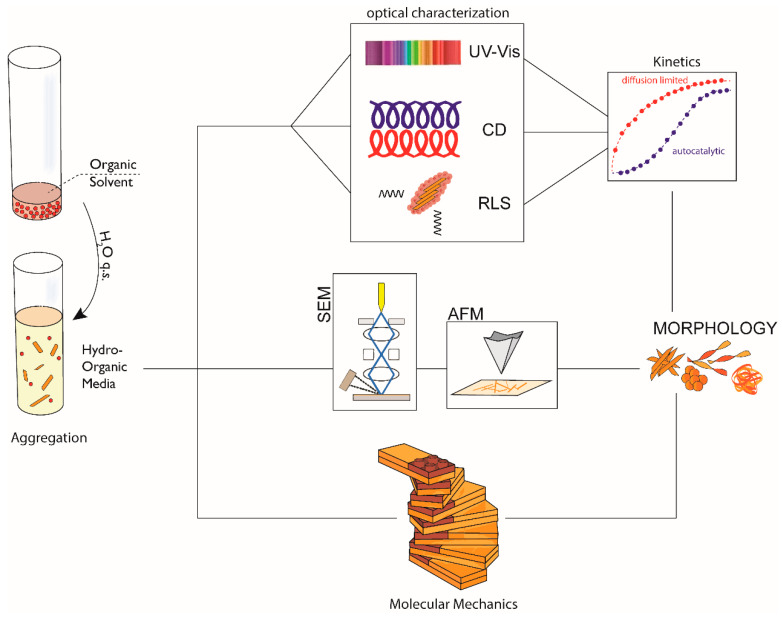
Schematic cartoon illustrative of our general approach for the aggregation studies.

In our work, we typically study the whole aggregation process and the possible formation of chiral porphyrin assemblies by combining different spectroscopic techniques, each contributing to elucidate on a distinctive aspect of the overall aggregative process (Figure 1). As UV-Vis and fluorescence spectroscopies give indications on the formation of aggregated species, whose arrangement in either specific or not specific geometries can be acquired from the changing of their typical spectral pattern (hypsochromic, bathocromic and hypochromic effects of the Soret band), Resonance Light Scattering spectroscopy studies provide information on the number of porphyrin units in electronic coupling constituting the formed assemblies, giving an estimation of the aggregate size. Circular dichroism is fundamental to highlight the chiral features of the aggregates, further revealing the mutual orientation (clockwise or counter-clockwise) of the porphyrin units within the supramolecular architecture. The kinetic of the aggregation can be usually followed by each one of these spectroscopies, enabling the outline of the intimate mechanism by fitting the experimental kinetic data with equations used for well-established models. The combined use of these spectroscopic techniques, sometimes supported by morphological analysis by AFM or SEM microscopies, allow us to assess: (i) if aggregation occurs, for example, to give, for example, J- or H-type aggregates, or aspecific species; (ii) the approximative size of porphyrin electronically coupled assemblies by RLS signals; (iii) if the supramolecular architectures formed possess an element of chirality and the mutual anticlockwise or clockwise arrangement of the chromophores forming the aggregates, with the help of the excitonic theory; (iv) the mechanism whereby the self-assembled species are formed and the effect of the variation of the bulk conditions. Indeed, it was frequently found that, for a given porphyrin substrate, the mechanism can vary by increasing porphyrin concentration or ionic strength, moving for example from an autocatalytic to a diffusion controlled pathway.

To support the gained experimental data, computational studies can be carried out, as semiempirical calculations or molecular mechanics, aiming to give further information on the structures of the final aggregates and, most importantly, on the driving force steering the specificity of the self-recognition process. By the concurrent use of all the described investigations and the rational collage of the gained information, the self-aggregation process can be described in all its aspects, enabling to further expand similar studies to other planned derivatives.

## 3. “Sweet” Porphyrins: The Solvent-Driven Aggregation of Glycosylated Porphyrin Derivatives

The idea of combining porphyrins and sugar molecules goes back to nearly 40 years ago in response to the need to dampen the hydrophobic character of the porphyrin dyes that prevents their solubility in physiological fluids and, consequently, their efficacy as therapeutic agents in cancer treatments. Besides the increased amphiphilicity, the incorporated sugar residue favors the targeting uptake and subcellular localization of these conjugated systems, being recognized by cell surface carbohydrate receptors expressed in tumor cells. For these reasons, a huge number of glycosylated porphyrins and their possible use in diagnostics and therapeutics have been reported over the years, particularly focusing on the photodynamic therapy field [43]. From a synthetical point of view, the incorporation of sugar moieties into porphyrin scaffold usually follows two paths: (i) preparation of suitable glycosylated aldehydes and their reaction with pyrrole under traditional experimental conditions leading to the porphyrin macrocycle and (ii) post-functionalization of porphyrin platforms having functional groups (-OH, -COOH, -CCH groups, etc.) suitable to react with proper sugar derivatives [44,45,46,47,48,49,50].

**Scheme 1:**
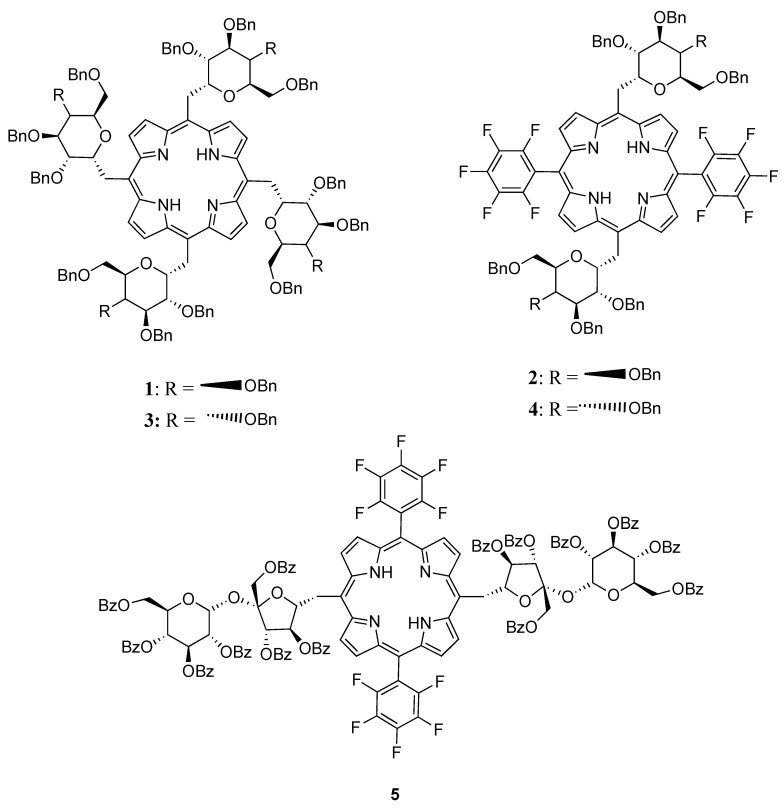
Glycoporphyin conjugates (**1**–**5**) employed in the aggregation studies thoroughly reported in References [34,35,38].

Following the first road, we have prepared a series of porphyrin–carbohydrate conjugates with “sugar” moieties directly placed in the meso-position via robust covalent C-C links [34,35,38], showing more stability towards hydrolytic or enzymatic degradation relative to the *O*-glycosidic bonds generally used (Scheme 1). The choice of the appended, polar sugars in combination with the lipophilic pentafluorophenyl substituents (C_6_F_5_) was dictated by the will to finely tune the amphiphilic properties of the porphyrin platforms and, thus, their aggregative behavior in aqueous media. More importantly for our purposes, the presence of stereogenic centers on these molecules and the straightforward access to diastereomeric couples (**1**–**3** and **2**–**4** pairs, respectively) proved to be fundamental for the construction of asymmetric porphyrin architectures with tunable and specular chiral properties.

In detail, glycoporphyrin derivatives **1**–**4** differ in their peripheral substitution pattern on the number as well as the type of appended “sugar” moieties (d-galacto and d-gluco functionalities for **1**–**2** and **3**–**4**, respectively). For a given monosaccharide, four or two meso-positions are occupied by sugar, the latter being alternated with two pentafluorophenyl rings in compound **2** and **4**. The derivatives were obtained starting with the proper “sugar”-aldehyde, i.e., 2-(2,3,4,6-Tetra-*O*-benzyl-β-d-galactopyranosyl)-ethyl aldehyde, which was condensed with pyrrole in one case under the Lindsey conditions to give tetrasubstituted porphyrin **1**. In the other, **2** was achieved according to the 2 + 2 MacDonald procedure, where pyrrole and aldehyde formed the dipyrromethane intermediate, later condensed with pentafluorophenylbenzaldehyde.

Glycoporphyrins **1**–**4** showed good solubility both in chlorinated and polar aprotic solvents like acetonitrile, dimethylacetamide, dimethylformamide and dimethyl sulfoxide. The UV-Vis spectral features of these compounds in chloroform presented a very sharp Soret band centered at 422 and 416 nm for the **1**–**3** and **2**–**4** analogues, respectively, with the observed hypsochromic shift for disubstituted compounds, explainable in term of electron-withdrawing effect exerted by the C_6_F_5_ groups.

**Figure 2:**
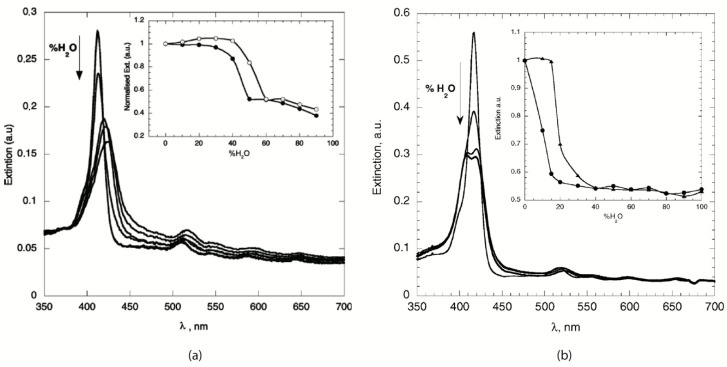
Spectral variations of glycoporphyrins increasing water proportion. (**a**) Porphyrin **2** (1.3 μM) in CH_3_CN-H_2_O solvent mixtures. Inset: aggregation profiles for **1** (lower curve) and **2** (upper curve) 3.7 μM, at T = 298 K; (**b**) porphyrin **4** (3.4 μM) in EtOH/H_2_O mixtures. Inset: aggregation profiles for **3** (lower curve) and **4** (upper curve). Adapted with permissions from [34,35]. Copyright 2007 and 2008, The Royal Society of Chemistry.

Going into the detail of the aggregation behavior of porphyrins **1** and **2**, it was found that CH_3_CN-H_2_O solvent mixtures of proper water content enable the monitoring of the aggregation phenomenon by conventional spectroscopic studies by UV-vis, fluorescence, and RLS techniques. Figure 2a reports the aggregation profile in CH_3_CN-H_2_O for compound **2**, ongoing through 0 to 100% water composition.

**Figure 3:**
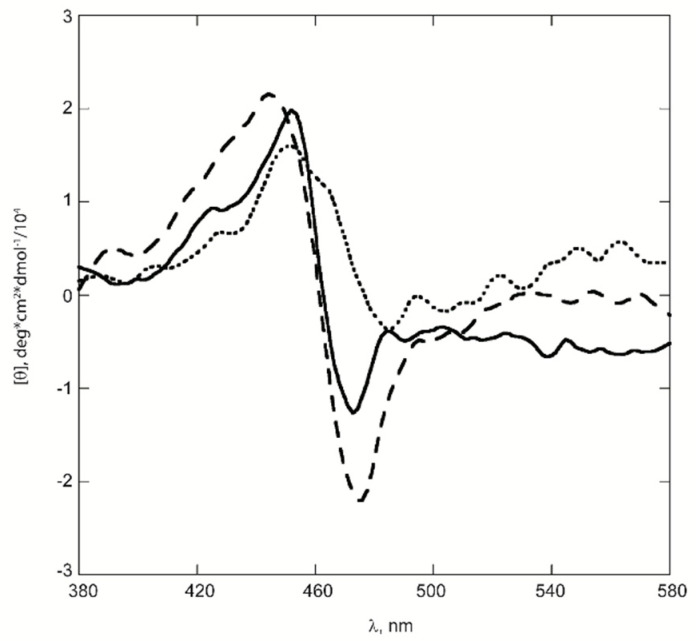
CD spectra of porphyrins **1** and **2** (3.7 μM) in aggregative conditions. (Solid line) **1** in H_2_O/CH_3_CN 50:50; (dashed line) **2** in H_2_O/CH_3_CN 55:45; (dotted line) **2** in H_2_O/CH_3_CN 55:45, NaBr 0.010 M. Adapted with permission from [34]. Copyright 2007, The royal Society of Chemistry.

The hypochromic effect together with the red-shift of the Soret band clearly indicates the formation of aggregated species with J-type, edge-to-edge, arrangement by hydrophobic interactions between the tetrapyrrolic platforms triggered by water increasing content (Figure 2a). As showed in Figure 2a (inset), some differences in the aggregation behavior were found for the two compounds. Namely, the critical aggregation composition (*cac*) occurs in the case of compound **2** at higher water proportion, likely due to the presence of the fluorine atoms, which should be involved in more efficient H-bonds. Fluorescence experiments confirmed the formation of porphyrin aggregates, since a strong fluorescence quenching upon increasing water proportion was evident for the two macrocycles. RLS studies further corroborated the occurrence of the aggregation phenomena, showing a gradual growth of the RLS signal on the Soret band region of the aggregates (*ca* 430 nm), with a maximum value reached above 60% H_2_O for porphyrin **1**. The RLS signal intensity led us to conclude that the formed aggregates are typically constituted of 10^4^–10^5^ porphyrin units in electronic conjugation. Remarkably, by comparing the RLS intensities for aggregates based on porphyrin **1** and **2**, we observed that aggregates with a lower degree of electronic coupling were obtained in the latter case. Kinetic experiments, carried out by following the decrease in the Soret bands with time, allowed for the elucidation of the intimate mechanism of formation of the supramolecular self-assembled structures [†]. In the case of both 1 and 2 derivatives, the experimental data were properly fitted by a “stretched exponential” equation, stating that the initial smaller clusters (nucleation seeds) “glob up” randomly monomeric units, forming larger aggregates (DLA, Diffusion Limited Aggregation mechanism) [51]. However, the different structural parameters are reflected in different aggregation rate constant and *n* values (where *n* is the aggregation growth factor, related to the mean value of binding sites available for aggregate growth process that is required to be <1 in the case of DLA mechanism); indeed, C_6_F_5_ groups confer a more favorable solvation to the porphyrin 2, which self-aggregate more slowly and with lower mean *n* values with respect to 1. The effect of ionic strength on the aggregation process has been also examined, carrying out the aggregation experiments at different concentration of NaBr on the bulk medium. The process resulted faster for both compounds without significative changes in the *n* values, suggesting an aspecific, general salt effect on the self-interaction events between the macrocycles, which aggregate more quickly but in smaller suprastructures, as evidenced by the reduced scattering intensities in RLS spectra. CD spectroscopic measurements gave an indication of the chiral properties featured by the self-assembled species formed in the different experimental conditions (Figure 3). For both compounds, the aggregation in CH_3_CN/H_2_O mixtures of proper water content resulted in strong, negative bisignate CD spectrum, indicating that porphyrins arrange in an anticlockwise (−) mutual conformation in the aggregates, according to excitonic theory [52]. Notably, a stronger CD signal was observed for the assemblies formed by porphyrin 2, which gave structures of smaller size but characterized by a higher degree of asymmetry (Figure 3, dashed line). Furthermore, the addition of NaBr had a strong impact on the chiroptical properties of the formed aggregates, which displayed CD bands of lower intensities (Figure 3, dotted line). This finding can be plausibly explained in terms of the shielding effect of the Na^+^ with the polar groups on porphyrin macrocycle, hampering an effective reading of the chiral information during the molecular recognition event.

**Figure 4:**
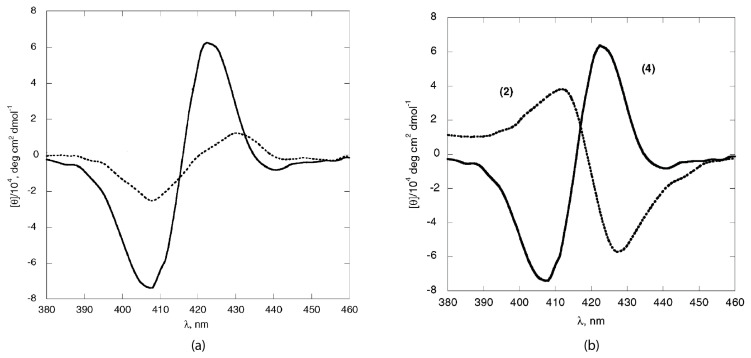
CD spectroscopic features of glycoporphyrins in EtOH/H_2_O mixture: (**a**) porphyrin **4** (3.4 μM) in 40% EtOH (solid line) and EtOH 40%, NaBr 0.074 M (dashed line); (**b**) porphyrin **2** and **4** in EtOH 40%. Adapted with permission from [35]. Copyright 2008, The Royal Society of Chemistry.

Preliminary experiments carried out in CH_3_CN-H_2_O media for compounds **3** and **4** showed, with respect to the d-galacto **1** and **2** species, the formation of H-type aggregates, as witnessed evident by the blue-shifted Soret bands. However, kinetic studies were carried out in more “halophilic” EtOH/H_2_O media, owing to the better solubility of the substrates **3** and **4**, at micromolar concentration, in the new solvent conditions. As shown in Figure 2b, aggregation of the title porphyrins in hydroalcoholic mixtures again occurred with the formation of H-type aggregates, at critical solvent composition of 80% and 85% ethanol content for **3** and **4**, respectively. [œ] Fixing the same solvent composition for both compounds at 85% ethanol, kinetic studies were carried out and *k* and *n* parameters were obtained by applying the “stretched exponential “equation. Even the self-aggregation of **3** and **4** relied on the DLA mechanism, with a slower formation of aggregates for **3** with respect to **4**, probably ascribable to the presence of the C_6_F_5_ groups that promote the sugar-π. Additionally, in this case, salt addition favored the aggregation of both compounds, remarkably affecting the aggregate morphology resulting in less specific suprastructures. Notably, in the case of porphyrin **4**, the *n* factor increased from 0.16 to 0.35 in the salt-promoted aggregation, indicating a change in the aggregation mechanism from DLA to DLCCA (Diffusion Limited Cluster-Cluster Aggregation), where the aggregate grows by collapsing of smaller pre-formed clusters rather than by further monomer inclusion [53]. Importantly, regarding the chiroptical features of the formed aggregates, intense positive, bisignate CD bands were found for both the d-gluco derivatives (Figure 4a, solid line, porphyrin **4**). This indicates that sugar moieties drive the aggregation toward the formation of porphyrin architectures, where chromophores are held in a mutual clockwise (+) chiral orientation. On the other hand, aggregates formed with NaBr addition displayed a strong decrease in the CD bands’ intensity, showing somewhat bathochromically shifted spectral features: in that event, the effective reading out of the chiral information during the aggregation was hampered by sodium cations that shield the sugar groups (Figure 4a, dashed line, porphyrin **4**), as previously found for the d-galacto analogues. Remarkably, Figure 4b stresses the chiroptical properties of opposite sign displayed by the two sets of glycoporphyrins investigated **1**–**2** and **3**–**4**, which are strictly related to the configurational change at C4 of the sugar moieties steering the overall self-aggregation process. Thus, we demonstrated that, by properly tuning the configuration of the stereogenic center of the peripheral groups on the porphyrin framework, it is possible to modulate the overall supramolecular chirality of the final architectures.

With this finding in mind, we recently studied how the particular molecular structure of the selected “sugar” moiety can influence the self-aggregation behavior of porphyrin–sugar conjugates in aqueous media. For such a purpose, porphyrin **5** with a more complex sugar group appended on the 10- and 20-*meso* positions (i.e., sucrose) was synthesized and preliminary results on its self-assembly attitude were directly compared with the A_2_B_2_ porphyrin analogues **2** and **4** [38]. As a main outcome, we found that **5** formed aggregated species in dimethyl acetamide (DMAC) or dimethylformamide (DMF)–water mixtures (3:2, *v*/*v*), featuring unexpected CD spectra. Indeed, only one positive, red-shifted, peak was observed by CD spectroscopic measurements, determined by THE induced Cotton effect of the chiral disaccharide on a single porphyrin molecule (ICD). This finding was completely different from the “d-galacto” and “d-gluco” porphyrin derivatives suprastructures that showed strong, bisignate CD spectra. Based on this, we can assert that the transfer of chiral information from disaccharide to the whole assembly is not effective, being short-range and locally operative because of the population of different conformational structures. Once more, it can be demonstrated that the choice of the ancillary sugars linked to the periphery of the porphyrin macrocycles is crucial for the construction of asymmetric supramolecular architectures.

## 4. “Fatty” Porphyrins: The Self-Assembly of Steroidal Porphyrins

**Scheme 2:**
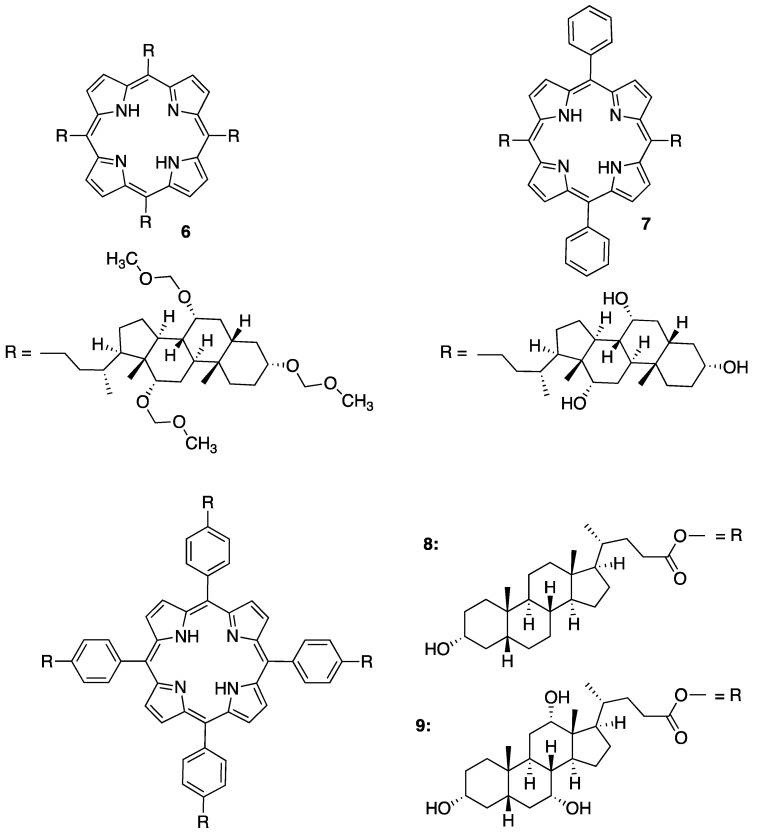
Steroidal porphyrin derivatives (**6**–**9**) investigated in the aggregation studies reported in References [40,41,42].

Over the years, we were involved in the self-aggregation studies of different steroidal porphyrin derivatives (Scheme 2), owing to their importance in different applications spanning from anion sensing and molecular recognition to targeted photodynamic treatments, where they can bind to tumor expressed saccharides [54,55,56,57,58,59]. For this purpose, the understanding of the aggregative behavior of these compounds in aqueous media fairly close to the physiological environments can be particularly important. As a first investigation, we reported on the aggregation properties of the tetrasteroid porphyrin derivative 6 in aqueous organic solvent mixtures [40].

**Figure 5:**
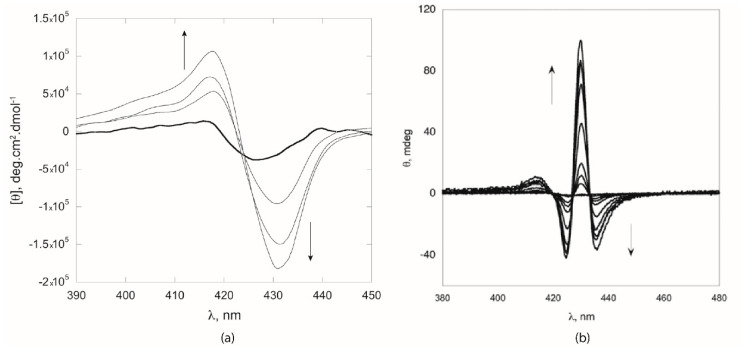
CD spectroscopic features of steroid-functionalized porphyrins **6** and **7**: (**a**) porphyrin **6** in dimethyl acetamide (DMAC)/H_2_O (84/16 *v*:*v*) mixtures upon increasing porphyrin concentration from 1.2 to 4.5 μM; (**b**) spectral variation of porphyrin **7** (3.0 μM) with time in DMAC/H_2_O (58/42, *v*:*v*) Adapted with permissions from [40,41]. Copyright 2013, World Scientific Publishing Company and Copyright 2014, The Royal Society of Chemistry.

Its amphiphilic character was provided by the three polar oligooxamethylene chains for each steroidal unit located at the meso-position, promoting suitable solubility in mixed aqueous solutions. The aggregation studies have been efficiently performed in dimethyl acetamide (DMAC)/H_2_O as well as dimethyl sulfoxide (DMSO)/H_2_O mixtures, highlighting an interesting influence of the organic solvent on the mechanism involved in the formation of the self-assembled structures. A different solvation effect of the two hydro-organic media reflects on the critical solvent composition at which the aggregation occurs. Indeed, the aggregation of porphyrin **6** in DMAC/H_2_O is complete within 20% of water, with formation of J-type aggregates with the Soret band occurring at 427 nm, while in DMSO/H_2_O the aggregated species are formed earlier at 15% of H_2_O content, featuring the Soret band at 430 nm. CD spectroscopy evidenced the chiral features distinctive of these suprastructures, giving in both solvent mixtures clear bisignate, negative, dichroic bands, indicative of counter-clockwise mutual arrangement of the electronically coupled chromophores (M-configuration) (Figure 5a) [52]. Quite surprisingly, we found that the stereochemistry of the aggregation process was not affected by the different solvation properties of the media, which, conversely, deeply influenced the rate and mechanism of the aggregates formation. Moreover, a remarkable effect of the starting porphyrin concentration on the mechanism involved was highlighted. Indeed, detailed investigations of the aggregation kinetics of porphyrin 6 carried out in DMAC/H_2_O 84/16 (*v*:*v*) at low concentration range spanning from 1.25 to 3.15 μM, revealed a typical sigmoidal profile with an initial lag time, indicative of fractal-type species grown by a cooperative, autocatalytic mechanism [60]. Remarkably, on further increasing the porphyrin concentration up to ca. 4.0 μM, a change toward a DLA mechanism was observed. In this case, we can surmise that the initial rapid formation of fractal clusters was followed by the slow inclusion of porphyrin monomers, the latter being more favored at a high monomer concentration. Different results have been obtained by investigating the self-aggregation of porphyrin 6 in DMSO/H_2_O 92/8 (*v*:*v*), where a DLA behavior was found to operate within the whole concentration range investigated (i.e., 1.4–3.8 μM). Additionally, the variation of substrate concentration in this case also induced a change toward a different type of mechanism, witnessed by a reduction of the aggregation stretching parameter *n* from 0.42 to 0.27 in correspondence with the 2.16–3.78 μM range (DLCCA model) [53]. This finding indicated that the suprastructures grew by the collapse of fast-formed aggregated nuclei, rather than by increasing in size by monomer inclusion. Altogether, these investigations revealed that the aggregation is faster in the more polar solvent mixture DMSO/H_2_O, confirming the hydrophobic effects as the driving interactions fostering the aggregation process of this steroidal porphyrin derivative.

**Figure 6:**
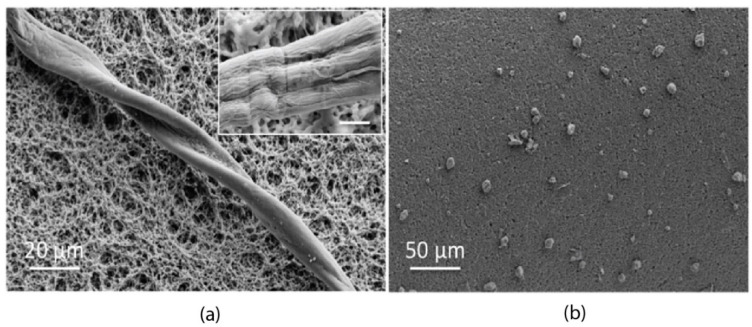
SEM images of porphyrin **7** self-aggregated in DMAC/H_2_O (58/42, *v*:*v*): c = 3.0 μM ((**a**), inset: enlargement of a structure obtained under the same conditions, scale bar 5 μm): (**b**) c = 10 μM. Adapted with permission from [41]. Copyright 2014, The Royal Society of Chemistry

The structural modification of porphyrin 6 to give porphyrin **7** (Scheme 2) enabled the formation of steroidal–porphyrin aggregates with different chiroptical and morphological properties [41]. These two derivatives differ for the presence of two trans-meso phenyl rings and two cholic-acid appended groups in the other meso-positions, where the previously oligooxamethylene chains are replaced by OH groups. Clearly, this structural change enhances the amphiphilicity of the derivative **7**, making the self-aggregation in aqueous media possible. The title porphyrin was found to form J-type aggregated structures when aggregating in DMAC/H_2_O 58/42 (*v*:*v*) solvent mixtures, as evidenced by the red-shift of the Soret band from 417 to 430 nm, going from the state of monomer to aggregate, respectively. Kinetic studies were carried out at this solvent composition, varying the porphyrin concentration range from 3 to 10 μM. A remarkable change in the aggregation mechanism was found also in this case, strictly dependent on the initial concentration of the macrocycle. Indeed, at low concentration (3–5 μM range) a biphasic behavior was observed, characterized by an initial aggregation process followed by a slower step, where macrocycles self-assembled in the first-formed stage undergo a structural rearrangement. The increase in porphyrin concentration up to 10 μM had a remarkable effect on the mechanism, which became controlled by diffusion (DLA mechanism). In this case, the higher concentration favors the rapid formation of aggregation nuclei, probably constituted by few monomers that slowly evolve into larger suprastructures. CD spectra recorded at different concentrations showed that aggregation occurs with formation of chiral assembled species featuring a peculiar spectral pattern, remarkably different from that observed for chiral systems based on tetrasteroid-porphyrin **6**. As showed in Figure 5b, the overall CD spectrum derives from the superimposition of two couple bands, with the more intense at 426 nm showing a −/+/−/+ sign and the other, less intense, at 420 nm with a −/+ profile. This peculiar pattern was recently observed by us in chiral assemblies formed by self-aggregation of a cationic Zn(II) (l)-prolinated tetraarylporphyrin derivative [33] and was illustrative of porphyrin suprastructures with rod-like morphology, in which the J-aggregate species are excitonically coupled along preferential space direction. Interestingly, by monitoring the aggregation process by CD spectroscopy, it was found that the dichroic signal emerges only after a first induction period, pointing out the initial formation of aspecific and non-chiral species that in the second slower stage, rearrange in species with chiral features. SEM images gave further insights on the morphology of the assemblies obtained, confirming the crucial effect of porphyrin concentration. Figure 6 shows the chiral species formed at 3 (right) and 10 μM (left) concentration, respectively. As a consequence of a change in the mechanism of self-assembly, different mesoscopic structures are obtained, in the form of large helicoidal architectures hundreds of micrometers long and *ca* 20 μm wide at lower concentration, and aspecific aggregates of globular shape with diameters spanning from 5 to 15 μm at a higher substrate concentration.

Molecular mechanics investigations highlighted the striking role of the hydrophobic effect, as well as the onset of steroidal OH-phenyl groups interactions during the self-assembling process. Furthermore, the energy minimization carried out on two right-handed helical decamers of derivative **7** indicated their spontaneous tendency to form a superhelix structure associated with an energy gain of about 53 kJ mol^−1^, which represents the driving force for the assembly of the mesoscopic structures reported in Figure 6.

Chiral mesoscopic structures based on tetrasteroidal porphyrin derivative **8** and **9** differing by the number of OH groups placed at the steroid rings (Scheme 2), have been also recently obtained by Langmuir–Blodgett (LB) deposition technique [42], which represents a promising and powerful strategy in interfacial nanoarchitectonics [61]. An effective transfer of chiral information from the porphyrin units to the suprastructures was here achieved at the air/water interface under increasing surface pressure conditions for both derivatives, with distinctive implications depending on their different amphiphilic character, modulated by the number of hydroxy groups in the steroid units. Interesting insights on the molecular organization of the two porphyrin LB films deposited on hydrophilic quartz surface were revealed by CD measurements. Indeed, a specular shape of the **8** (+/−) and **9** (−/+) dichroic bands was observed, with almost the same intensity, indicating the formation of porphyrin-based helical suprastructures of similar size, but opposite chirality. Furthermore, atomic force and scanning electrons microscopies revealed that these aggregates arranged in twisted ribbons or fibers for **8** and **9**, respectively. Molecular Mechanics (MM) calculations highlighted the structural motifs governing the hierarchical assembly of these steroidal derivatives: (i) the clockwise or anti-clockwise winding of the steroid chains that transfers the chiral information of the steroid groups from the molecular to the higher organization level of the helically-arranged porphyrin systems, and (ii) the amphiphilic character of the outer surface of these aggregates controls their assembly in different mesoscopic structures. Of course, the sole hydrogen bond interactions cannot explain the different behavior observed in the arrangement of the two derivatives; reasonably, it would depend on the steric hindrance of the steroid chains during the growth of the porphyrin fibers as well as the different amphiphilic character of the surfaces of the self-assembled structures. Indeed, because of a strong amphiphilicity, the helical fibers derived by **9** organize themselves in mesoscopic rods constituted by strictly entwined multifibers. Conversely, mesoscopic aggregates based on porphyrin **8** display a twisted ribbon morphology resulting from hydrophobic effects driving the self-assembly of molecular building blocks.

## 5. “Sugars” and “Fats” on the Same Molecule: Who Determines the Final Supramolecular Chirality? The Case of Glucosylated Steroid-Porphyrins

**Scheme 3:**
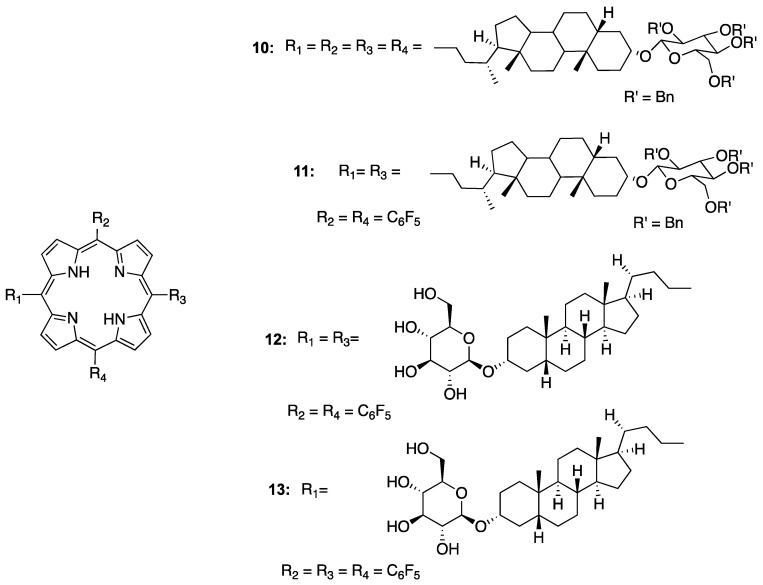
Glucosylated steroid–porphyrin conjugates (**10**–**13**) investigated in the aggregation studies reported in References [36,37].

The results already described show that the peripheral chiral ancillary groups (sugars and steroids) bound to the porphyrin ring drive the formation of supramolecular structures with a high degree of asymmetry, with morphological features strictly depending on the stereogenic moieties placed on the porphyrin building block. As a further extent of these studies, four glucosylated steroid–porphyrin derivatives, differing in the number and type of ring substitutions, were investigated by optical spectroscopy methods and reported in two subsequent works about ten years ago [36,37]. Their molecular structures and numbering are given in Scheme 3. This particular functionalization offered the possibility to explore the combining effects of three components, namely the aromatic porphyrin platform, steroidal and glucosyl moieties, on the chiroptical and structural properties of the final assemblies. Additionally, the concomitant presence of the two chiral functionalities within the same macrocycle allowed us to assess a possible predominance of one group on the other in driving the stereospecificity of the aggregation process. Going into detail, we firstly investigated the self-aggregation in DMAC/H_2_O in various proportions of the porphyrin derivatives **10** and **11**, characterized by the presence of four or two “*glucosteroid*” substituents in the *meso*-position, respectively. In the case of 10, the aggregation was complete with 20% of water, whereas for the more polar **11** complete aggregation required a larger fraction of water (40%). The different spectroscopic techniques used evidenced a different aggregative behavior for the two derivatives, clearly ascribable to the diverse steric hindrance due to the bulky steroid pendants, being smaller for derivative **11** with respect to **10**. Indeed, UV/Vis spectroscopy revealed that aggregation of porphyrin **10** at DMAC 80% proceeded with the formation of J aggregates, with scarcely red-shifted bands, to whom a blue shifted band at 405 nm emerged at a higher porphyrin concentration, indicating the coexistence of mixed J- and H-type morphologies.

Conversely, in the case of the disubstituted derivative **11**, the self-aggregation gave species featuring excitonically coupled Soret bands, having a spectroscopic barycentre fixed at 413 nm. CD spectroscopic measurements were consistent with the UV/Vis results, showing that in all the cases the aggregation led to chiral assemblies but with remarkably strong differences in terms of band intensity and shape. Indeed, in the case of porphyrin 10 different CD spectra were recorded varying the aggregative conditions. At ca. 1 μm in pure DMAC, a weak dichroic negative band at 419 nm was evident, originating from both the induction effect of the chiral substituents (ICD) and the effect of distortion of the porphyrin ring from planarity. In aggregative conditions (>30% H_2_O), a very weak signal was detected ascribable to an aspecific, scarcely optically-active species formation. Finally, at higher porphyrin concentration both higher-energy and lower-energy, partially superimposed, bisignate negative bands were observed in this case, indicating also the concurrent formation of H- and J-type chiral species. Interestingly, the CD spectral profile of the porphyrin **11** aggregation evolved in a different way, going from the induced negative band in pure DMAC to a strong, negative bisignate Cotton effect when DMAC proportion was 40%, suggesting the anticlockwise arrangement of the porphyrin platforms within the formed suprastructures. It is noteworthy that the CD spectra of these species featured bands of opposite sign to that observed for simpler glycosylated porphyrin **3** and **4**, lacking the steroidal moieties. On the basis of these results, we have concluded that the mutual spatial configuration of the macrocycles was certainly governed by the chiral information borne by the rigid steroid pendants, with the sugar-appended units irrelevant to the stereospecific recognition event between the building blocks. Computational studies on aggregated structures of the disubstituted derivative **11** also corroborated this experimental result, showing that the favorable van der Waals interactions between the steroid pendants were responsible for the highest stability of the anticlockwise isomer of M helicity and governed the handedness of higher aggregates.

**Figure 7:**
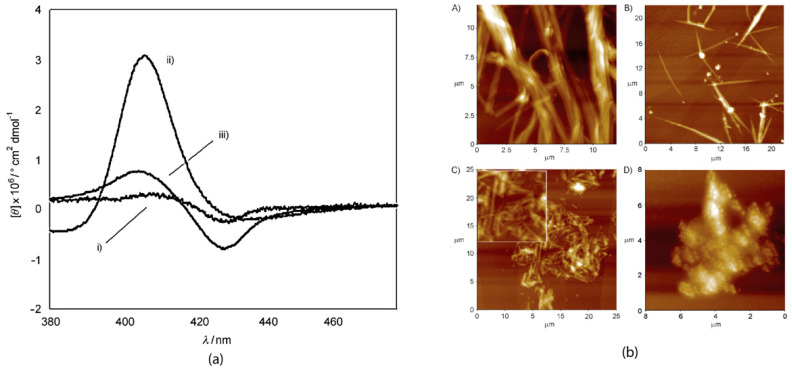
(**a**) CD spectra of porphyrin **11** in DMAC/H_2_O (87/13 *v*:*v*) mixtures at different porphyrin concentrations: (i) 0.8, (ii) 1.6, (iii) 2.4 μM; (**b**) Corresponding AFM images (HOPG) of aggregates of **11** formed in the same conditions: (A) 0.8 μM; (B) 1.6 μM; (C) 2.4 μM (inset: an enlargement of the central areas); (D) AFM images of **10** aggregates (2.4 μM, DMAC/H_2_O, 91/9). Adapted with permission from [36]. Copyright 2011, Wiley-VHC Verlang GmbH & Co KGaA. Weinheim.

Parallel kinetic investigations gave information on the aggregation mechanisms for both derivatives. The self-aggregation of porphyrin 10 investigated at DMAC/H_2_O 91/9 (*v*/*v*) proportions occurred following the DLA mechanism on the whole concentration range explored (0.7–3.3 μM), showing a specific value of 2.7 μM after which the *k* rate constant did not increase with the concentration. This finding was in line with the UV/Vis results that evidenced the formation at this concentration value of mixed population of J- and H-type aggregates. A more elaborate process was assessed for the aggregation of 11 in DMAC/H_2_O 87/13 (*v*/*v*) mixture. Indeed, at 0.8 μM an autocatalytic mechanism was found with the formation of fractal-type species. A progressive change of *n* and *m* parameters was observed with an increase in porphyrin concentration to 1.6 μM, revealing a gradual biasing toward a mechanism forming larger nucleation seeds that grow in a second step characterized by a reduced level of cooperativity (*n* decreased from 7 to *ca* 3). Increasing the initial porphyrin concentration up to 2.4 μM, a complete kinetic change toward the DLA mechanism was observed. CD measurements confirmed the kinetic results by the final ellipticity of the CD bands recorded at different concentrations: indeed, the maximum *θ* values increased by one order of magnitude going from 0.8 to 1.6 μM, while the further increase in concentration until 2.4 μM strongly decreased the CD band intensity, corroborating the observed change in mechanism (Figure 7a). These interesting findings were likewise confirmed by AFM studies, which were carried out by drop-casting aggregate solutions at the same solvent composition used for kinetic studies on freshly cleaved highly oriented pyrolytic graphite (HOPG) (Figure 7b). If almost aspecific randomly aggregated species were observed for aggregates of porphyrin 10 (Figure 7b, D), different aggregate morphologies have been detected for 11 at different concentrations, in accordance with the kinetic results. Namely, fibril structures handled in larger fibers have been found at 0.8 μM, while at intermediate concentration (1.6 μM) the structures were wider mostly isolated rods about 10 μm in length. Finally, passing at the higher concentration value of 2.4 μM, the observed structures were aggregate rods resulting from the coalescence of smaller globular entities into longer fibril-like structures (Figure 7b, A–C).

**Figure 8:**
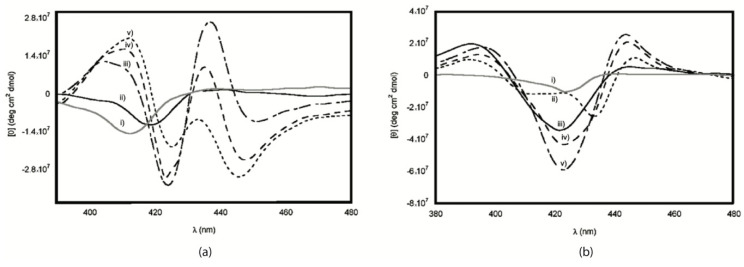
CD spectra of glucosylated steroid-porphyrins **12** (**a**) and **13** (**b**) (5 μM) in DMSO/H_2_O at increasing water content: a. 0%; b.20%; c. 30%; d. 40%; e. 50% Adapted with permission from [37]. Copyright 2012, Royal Society of Chemistry.

A further slight modification on the porphyrin peripheral substitution led to the two glucosylated steroid-porphyrins 12 and 13 that were investigated by optical spectroscopy methods with respect to not only their aggregation properties in solution, but also to their organization in chiral LB films onto hydrophilic quartz surface and their inclusion in a liposome formulation [37]. Their behavior in aggregative conditions was investigated in DMSO/H_2_O 40:60 (*v*/*v*). For both compounds, the red-shifted UV/Vis absorptions indicated the formation of J-type aggregates, characterized by a head-to-tail arrangement of the porphyrin monomers. CD measurements revealed significant differences in the dichroic spectra of the two derivatives. Indeed, while the CD spectrum of porphyrin **12** showed a (+/−/+) CD signal in the 380–460 nm spectral region, in the case of monosubstituted derivative **13** a complex set of CD bands was recorded, with two partially overlapped exciton-split signals, two crossover points placed at ca. 420 and 440 nm, likely ascribable to H- and J-type aggregates, respectively, and negative (−/+) bisignate bands (Figure 8). The different shape and sign distinctive of the CD bands of the two conjugates were indicative of a different morphology for the mesoscopic structures obtained in the two cases, probably determined by a different mutual arrangement of the chromophores constituting the nucleation seeds during the early aggregation stage. An interesting remark can be stressed by comparing the supramolecular chirality possessed by the assemblies formed by **13** and the disubstituted porphyrin **11**. A complete inversion of the CD signal was in fact observed for the two compounds, indicating a different orientation of the porphyrin units within the final architectures. The structural diversity of the two derivatives, merely localized on the glucosydic region, confers them a different solubility as the water proportion in the solvents used suggests. In the case of the more hydrophilic porphyrin **13**, the 60% water content rules out the occurrence of stereospecific recognition between the appended deprotected sugars of single porphyrin units, being presumably fully solvated at this water content. On the other hand, the massive solvation of these glucosyl groups forces the rigid, hydrophobic steroidal moieties to adopt a conformation different from that assumed in the aggregates formed by porphyrin **11**. In this case, a solvent-controlled chirogenesis of the supramolecular architectures can be invoked, leading to assemblies with almost specular spectra, the chiral group being equal.

## 6. Conclusions

The synthetic versatility of the porphyrin platforms allows us to finely modulate their physicochemical properties, and in particular the amphiphilic character by introducing polar and lipophilic groups in the scaffold. This opportunity is extremely beneficial for the formation of ordered supramolecular structures in mixed organic-aqueous media of proper characteristics, which are exploitable in different areas of research [11]. The further implementation of chirality elements into the amphiphilic porphyrin frame widens the interest and nanotechnological applications of the fabricated chiral porphyrin suprastructures, as for example, in chiral recognition and stereoselective sensors fields. Among the numerous chiral groups that could potentially decorate the porphyrin periphery, bio-related molecules as sugars or steroids represent a good choice thanks to their well-established chemistry, conformational rigidity, biological affinity and, in some cases, commercial availability. In this contribution, we have summarized our studies concerning the solvent-driven aggregation of a florilegium glyco-, steroidal- and glucosteroidal-porphyin derivatives in different aqueous media, showing the possibility to form chiral mesoscopic structures of reproducible and tunable size. We have showed that the topology and asymmetry of the obtained structures can range from rods and fibers to molecular wires, by properly implementing: (i) the type, the number and symmetry of the appended groups on the *meso*-positions; (ii) the solvent bulk properties, i.e., type and relative ratio of hydro-organic solvent used, ionic strength and substrate concentration.

The monitoring and the full understanding of how macrocycles self-aggregate in solution is made possible by the extraordinary photophysical properties of porphyrin chromophores whose variation throughout aggregation can be easily followed by optical methods. The general approach used in our study is particularly relevant for the development of chiral materials that, coupled with nanotechnology, could be used in many important fields such as catalysis, nonlinear optics, materials science and sensors.

## Notes

[†] The aggregation kinetics followed by RLS spectroscopy gave similar results, in satisfactory agreement. [œ] Aggregation experiments carried out on the d-galacto porphyrin **2** in ethanol/water solvent mixtures showed a large broadening, and red-shift of the Soret band, similarly to that observed in MeOH/water media. This rules out a solvent effect on the molecular arrangement of porphyrin platforms (J- or H-aggregated species), confirming the key role of the configuration of the glucoside moieties on the aggregate geometry.
